# Novel roles of TMEM100: inhibition metastasis and proliferation of hepatocellular carcinoma

**DOI:** 10.18632/oncotarget.3954

**Published:** 2015-04-29

**Authors:** Dipeng Ou, Hao Yang, Dong Hua, Shuai Xiao, Lianyue Yang

**Affiliations:** ^1^ Department of Geratic Surgery, Xiangya Hospital, Central South University, Changsha, Hunan, China; ^2^ Department of Surgery, Xiangya Hospital, Central South University, Changsha, Hunan, China; ^3^ Liver Cancer Laboratory, Xiangya Hospital, Central South University, Changsha, Hunan, China

**Keywords:** TMEM100, HCC, metastasis, proliferation, prognosis

## Abstract

Transmembrane protein 100 (TMEM100) was activated by ALK1/TGF-β signaling. We found that TMEM100 was decreased in hepatocellular carcinoma (HCC) tissues and in highly metastatic cell lines. Overexpressed of TMEM100 inhibited invasion, migration and proliferation. Low levels of TMEM100 were associated with cirrhosis, tumor size, Tumor nodule number, TNM stage, BCLC stage, Edmondson-Steiner Stage and vein invasion. Furthermore, TMEM100 was an independent risk factor for overall survival (*P* = 0.03) and disease-free survival (*P* = 0.019). The current findings suggest that TMEM100 functions as a tumor suppressor in HCC metastasis and proliferation.

## INTRODUCTION

Hepatocellular carcinoma (HCC) is one of the most common malignant cancers in the world, particularly in China. [1] It ranks as the fifth most common malignancy and the second leading cause of cancer death worldwide, resulting in more than 695,900 deaths each year [2, 3]. Despite the improvement in treatment of HCC during recent decades, this disease continues to have a high mortality rate mainly due to late diagnosis and the lack of effective therapies, especially for advanced HCC [4, 5]. Hepatocarcinogenesis involves multiple steps including accumulation of genetic and epigenetic alternations of the hepatocyte genomes, eventually leading to malignancy development [6]. Cell cycle deregulation is a hallmark of tumor cells [7] and targeting the proteins that mediate critical cell cycle processes is an emerging strategy for the treatment of cancer [8]. In recent decades, various molecules have been reported to play a role in invasion and migration of HCC, such as miR-140-5p, miR-331-3p, Egfl7, RhoC, FBI-1, and HSF1 [9-14]. Although these findings represent significant progress in the field, the mechanisms underlying HCC metastasis are still largely unknown. Therefore, it is very important to identify novel tumor suppressor-genes to prevent postoperative recurrence and metastasis of HCC.

Transmembrane protein 100 (TMEM100) is a gene at locus 17q32 encoding a 134-amino acid protein with two hypothetical transmembrane domains (amino acids 53–75, 85–107) [15]. TMEM100 is well conserved in vertebrates and not structurally related to any known family of proteins in any species [16]. Somekawa S et al. have found that knockout of TMEM100 would lead to mouse embryonic death due to cardiovascular developmental disorders [17]. Frullanti E et al has illustrated that TMEM100 inhibited the proliferation of lung cancer cells [18]. Since HCC is the tumor with rich vascular, we speculate that TMEM100 also play an important role in HCC.

However, evidence for the function of TMEM100 in human malignancies is still limited, especially in the case of HCC. Therefore, we carried out the present study to determine the expression of TMEM100 in human HCC tissues as well as cell lines. Furthermore, the biological functions of TMEM100 in HCC were also elucidated *in vitro* and *in vivo*.

## RESULTS

### TMEM100 was significantly downregulated in human HCC tissues and HCC cell lines

**Figure 1:**
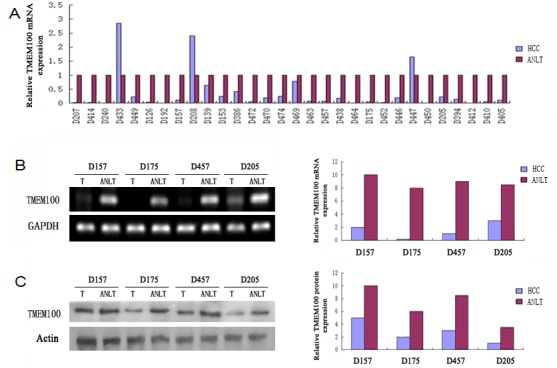
TMEM100 is frequently down-regulated in HCC tissues **A.** The mRNA expression levels of TMEM100 in 30 paired HCCs and non-HCCs were determined by quantitative real-time PCR and normalized by 2^−ΔΔct^ method, where GAPDH was used as internal control. Compared with noncancerous tissues, the mRNA level of TMEM100 was significantly lower in 90% HCC tissues as compared with that of ANLTs. **B.** The mRNA levels of TMEM100 in four matched HCC tissues and ANLTs were analyzed by semi-quantitative PCR.GAPDH was used as loading reference. **C.** The protein levels of TMEM100 in 30 matched HCC tissues and ANLTs were analyzed by western blotting. β-actin was used as loading reference.

**Figure 2:**
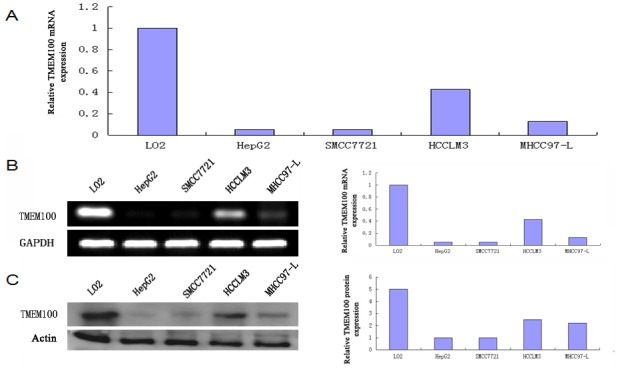
TMEM100 is also frequently down-regulated in HCC cell lines **A.** The mRNA expression levels of TMEM100 in five cell lines were determined by quantitative real-time PCR. **B.** The mRNA expression levels of TMEM100 in five cell lines were analyzed by semi-quantitative PCR. **C.** The protein levels of TMEM100 in five cell lines were analyzed by western blotting. β-actin was used as loading reference.

The results of qRT-PCR showed that the expressions of TMEM100 mRNA in HCC tissues were significantly lower than those in adjacent nontumorous liver tissues (ANLTs, Figure 1A). We select four HCC tissues, in which TMEM100 was relatively low expression at the mRNA level according to the qRT-PCR results, to detect TMEM100 mRNA and protein expression by RT-PCR and Western-blot. Our results showed that TMEM100 mRNA and protein expression level was significantly lower in HCC tissues compared to ANLTs (*P* < 0.05) (Figure 1B, 1C). Then, the expression of TMEM100 was also been detected in HCC cell lines and normal liver cells (L02 cell line). According to the data of qRT-PCR, the expression of TMEM100 mRNA in LO2 cell line was significantly higher than that in HCC cell lines including HepG2, SMMC-7721, HCCLM3 and MHCC97-L (Figure 2A). Furthermore, TMEM100 mRNA expression in the HCC cell line HepG2 and SMMC-7721 which with relative higher metastatic and proliferative potential [19], was very low (0.001 and 0.003), while in HCCLM3 and MHCC97-L which with relative lower metastatic and proliferative potential, TMEM100 mRNA expression was relatively high (0.47 and 0.03). Semi-quantitative RT-PCR and Western-blotting results were consistent with the Real-time PCR results (Figure 2B & 2C). From these data, we speculate that TMEM100 might inhibit the metastasis and proliferation of HCC cells and act as a tumor suppressor in HCC.

### Reduced expression of TMEM100 correlates with increased metastatic potential in HCC tissues

**Figure 3:**
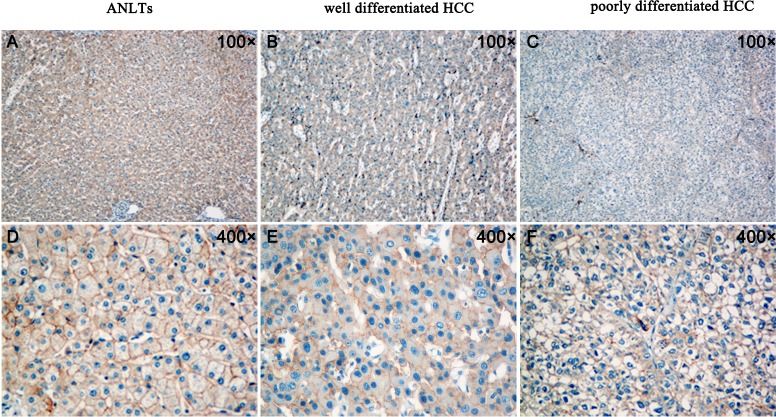
Analysis of TMEM100 expression in ANLTs, well differentiated hepatocellular carcinoma and poorly differentiated hepatocellular carcinoma by IHC **A.**&**D.** The expression of TMEM100 was strong positive in ANLTs (A:100×, D:400×). **B.**&**E.** The expression of TMEM100 was moderate positive in well differentiated hepatocellular carcinoma (B:100×, E:400×). **C.**&**F.** The expression of TMEM100 was week positive or negative in poorly differentiated hepatocellular carcinoma (C:100×, F:400×).

**Figure 4:**
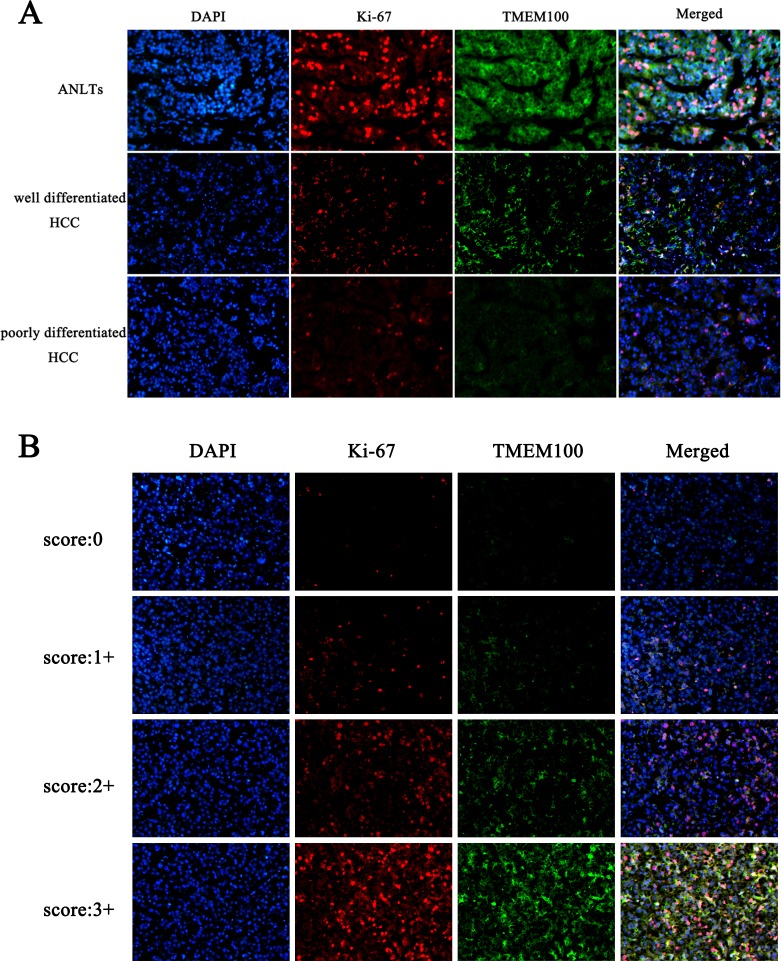
Double staining of TMEM100 and Ki-67 in HCC tissues **A.**-**B.** Immunohistofluorescence was applied to examine the expression of TMEM100 and Ki-67 in different differentiated HCC tissues. Our data showed that the expression of TMEM100 is consistent with Ki-67. Both of the expression of TMEM100 and Ki-67 were strong positive in ANLTs, moderate positive in well differentiated hepatocellular carcinoma and week positive or negative in poorly differentiated hepatocellular carcinoma.

To further verify the above hypothesis, we analyzed the expression of TMEM100 in 10 cases of ANLTs, well differentiated hepatocellular carcinoma tissues and poorly differentiated hepatocellular carcinoma tissues by immunohistochemistry, respectively. Our data found that the expression of TMEM100 was strong positive in 7 cases of ANLTs (7/10, Figure 3A & 3D), while the expression of TMEM100 was moderate positive in 6 cases of well differentiated hepatocellular carcinoma tissues (6/10, Figure 3B & 3E) and week positive or negative in 7 cases of poorly differentiated hepatocellular carcinoma tissues (7/10, Figure 3C & 3F). Furthermore, double staining with another malignancy-related marker (Ki-67) was also performed. Our data showed that the expression of TMEM100 is consistent with Ki-67. Both of the expression of TMEM100 and Ki-67 were strong positive in ANLTs, moderate positive in well differentiated hepatocellular carcinoma and week positive or negative in poorly differentiated hepatocellular carcinoma (Figure 4A & 4B). Of note is that the reduced expression of TMEM100 was in agreement with the metastatic and proliferative potential of these HCC tissues, suggesting a potential role for TMEM100 in HCC metastasis and proliferation.

### Down-regulation of TMEM100 is associated with HCC poor prognosis

**Figure 5:**
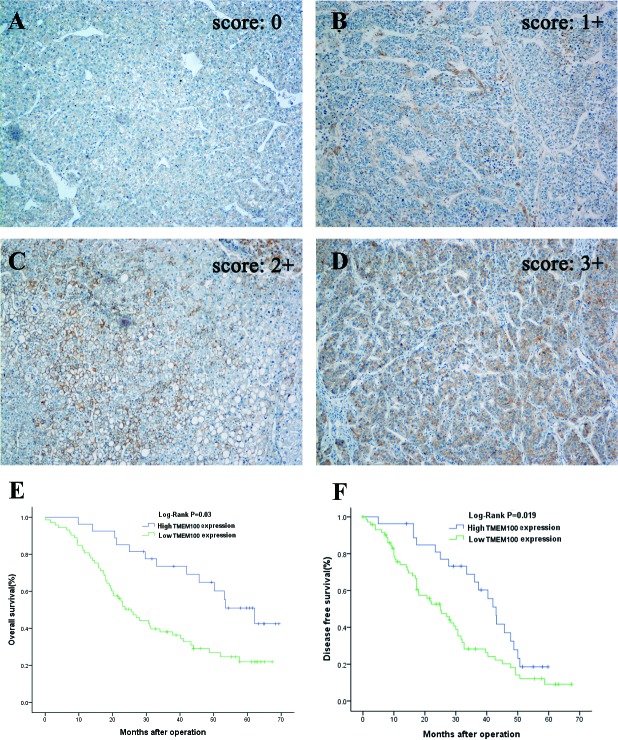
Immunohistochemistry of TMEM100 expression in HCC tissues and its prognostic implication **A.**-**D.** Immunohistochemistry was applied to examine the expression of TMEM100 in HCC tissues. In these representative images, the negative control **A.** is also included to show the specificity of the antibody, TMEM100 expression is seen in 11%-25% of cancer cells (scored as 1+, **B.**), 26%-50% of cancer cells (scored as 2+, **C.**) and more than 51% of cancer cells (scored as 3+, D). Original magnification 100×. **E**, **F.** Overall survival and disease-free survival was analyzed according to the expression of TMEM100 in 90 cases of HCCs (using the Kaplan-Meier method and evaluated using the log-rank test). The results showed that patients with HCC who had high TMEM100 expression have better overall survival than those with low TMEM100 expression (*P* = 0 .03). Analysis for disease-free survival shows the same results (*P* = 0.019).

To assess the feasibility of TMEM100 expression in HCC prognosis, analysis was conducted between two groups based on the immunohistochemistry results: one with relative low TMEM100 expressions (scored as 0 and 1+, n = 65, Figure 5A & 5B), while the other with relative high TMEM100 expressions (scored as 2+ and 3+, n = 25, Figure 5C & 5D). TMEM100 expression was significantly associated with cirrhosis (*p* = 0.03), tumor size (*p* < 0.001), Tumor nodule number (*p* < 0.001), TNM stage (*p* < 0.001), BCLC stage (*p* < 0.001), Edmondson-Steiner Stage (*p* = 0.002) and vein invasion (*p* < 0.001, Table 1), respectively. Next, the Cox proportional hazards regression model was introduced. On univariate survival analysis, cirrhosis (*p* = 0.012, *p* = 0.038), tumor nodule number (*p* = 0.019, *p* = 0.036), vein invasion (*p* = 0.024, *p* = 0.013) and TMEM100 expression (*p* = 0.008, *p* = 0.009) reached significance for overall survival (OS) and Disease-free survival (DFS), respectively (Table 2 & Table 3). On multivariate survival analysis, we found that the OS of HCC patients was significantly dependent on cirrhosis (*p* = 0.042), tumor nodule number (*p* = 0.014), vein invasion (*p* = 0.019) and TMEM100 expression levels (*p* = 0.021; Table 2), while DFS of HCC patients was significantly dependent on tumor nodule number (*p* = 0.028), vein invasion (*p* = 0.017) and TMEM100 expression levels (*p* = 0.026; Table 3). Furthermore, HCC patients with high TMEM100 expressions had much longer overall survival time (median survival time, 59.5 *vs.* 26.8 months, *p* = 0.030) than those with low TMEM100 expressions (Figure 5E). HCC patients with high TMEM100 expressions also had longer disease-free survival time (median survival time, 42.8 *vs.*24.1 months, *p* = 0.019; Figure 5F) than those with low TMEM100 expression.

**Table 1:** Correlations between TMEM100 expression level and clinicopathological variables of 90 cases of HCC in validation cohort

		TMEM100 expression		
Clinicopathologic Variables	N	Low	High	*P* value
Gender				
Male	72	52	20	
Female	18	13	5	1.000
Age(years)				
≤60	58	42	16	
>60	32	23	9	0.957
HBsAg				
Negative	28	20	8	
Positive	62	45	17	0.911
AFP				
Negative	36	24	12	
Positive	54	41	13	0.342
Cirrhosis				
Absence	31	18	13	
Presence	59	47	12	**0.030**
Child-Pugh Score				
A	29	14	15	
B	61	51	10	0.154
Tumor size (cm)				
≤5	27	13	14	
>5	63	52	11	**<0.001**
Capsular formation				
Presence	41	29	12	
Absence	49	36	13	0.776
Tumor nodule number				
Solitary	66	59	7	
Multiple(≥2)	24	6	18	**<0.001**
TNM Stage				
I/II	66	55	11	
III	24	10	14	**<0.001**
BCLC Stage				
0-A	60	51	9	
B-C	30	14	16	**<0.001**
Edmondson-Steiner Stage				
I-II	32	17	15	
III-IV	58	48	10	**0.002**
Vein invasion				
Presence	20	6	14	
Absence	70	59	11	**<0.001**

**Table 2:** The Cox regression analyses of overall survival (OS) and TMEM100 expression level as well as clinicopathological parameters

	Univariable analysis		Multivariable analysis	
Variables	HR (95% CI)	*P*	HR (95% CI )	*P*
Gender(Male *vs*. Female)	0.742 (0.313 - 2.126)	0.354		
Age(≤60 *vs.* > 60 )	0.687 (0.331 – 1.987)	0.441		
HBsAg(Negative *vs*. Positive)	0.924 (0.442 - 1.721)	0.424		
Cirrhosis(Presence *vs*. Absence)	2.776 (1.443 - 6.854)	**0.012**	1.921 (1.012 – 4.881)	**0.042**
Child-Pugh Score(Grade A *vs*. B)		1.652 (0.448 - 2.532)	0.664		
AFP level(ng/ml, ≤ 20 *vs*. > 20)	0.563 (0.198 - 1.822)	0.286		
Tumor size(>5 *vs*. ≤5 cm)	2.152 (0.916 - 2.762)	0.082	3.235 (0.942 – 3.678)	0.066
Capsular formation(Presence *vs*. Absence)	1.442 (0.486 – 2.665)	0.432		
Tumor nodule number(Solitary *vs*. Multiple)	3.112 (1.228 – 7.662)	**0.019**	4.652 (1.341 – 9.662)	**0.014**
Edmondson-Steiner Stage(I-II *vs*. III-IV)	1.325 (0.654 – 2.143)	0.358		
Vein invasion(Presence *vs*. Absence)	1.887 (1.264 – 2.838)	**0.024**	3.742 (1.132 – 7.785)	**0.019**
TMEM100 expression(Low *vs*. High)	2.451 (1.423 – 8.042)	**0.008**	3.942 (1.167 – 8.772)	**0.021**

**Table 3:** The Cox regression analyses of Disease-free survival (DFS) and TMEM100 expression level as well as clinicopathological parameters

	Univariable analysis		Multivariable analysis	
Variables	HR (95% CI)	*P*	HR (95% CI)	*P*
Gender(Male *vs*. Female)	0.912 (0.387 - 1.981)	0.513		
Age(≤60 *vs.* > 60 )	0.778 (0.342 – 2.711)	0.611		
HBsAg(Negative *vs*. Positive)	0.882 (0.621 - 2.629)	0.571		
Cirrhosis(Presence *vs*. Absence)	1.758 (1.241 - 7.642)	**0.038**	1.708 (0.898 – 4.824)	0.061
Child-Pugh Score(Grade A *vs*. B)	0.821 (0.392 - 2.231)	0.772		
AFP level(ng/ml, ≤ 20 *vs*. > 20)	0.736 (0.229 - 1.791)	0.338		
Tumor size(>5 *vs*. ≤5 cm)	1.847 (0.872 - 2.932)	0.119		
Capsular formation(Presence *vs*. Absence)	0.621 (0.423 – 1.118)	0.082	0.808 (0.232 – 1.635)	0.649
Tumor nodule number(Solitary *vs*. Multiple)	0.483 (0.288 – 0.896)	**0.036**	0.421 (0.244 – 0.985)	**0.028**
Edmondson-Steiner Stage(I-II *vs*. III-IV)	0.462 (0.311 – 1.276)	0.242		
Vein invasion(Presence *vs*. Absence)	1.586 (1.112 – 2.783)	**0.013**	1.898(1.087 – 3.824)	**0.017**
TMEM100 expression(Low *vs*. High)	2.128 (1.132 – 5.217)	**0.009**	2.612 (1.227 – 4.561)	**0.026**

### TMEM100 inhibits HCC metastasis and proliferation *in vitro* and *in vivo*

**Figure 6:**
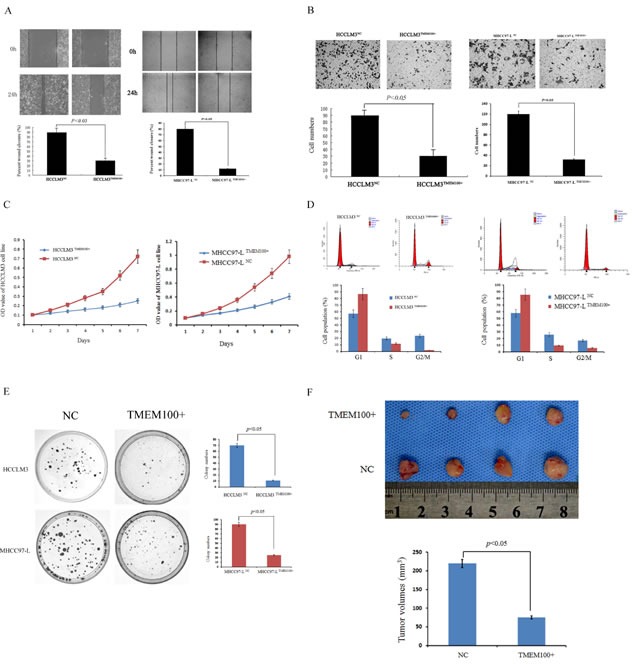
TMEM100 inhibits HCC cell migration, invasion, growth and colony formation *in vitro* and *in vivo* **A.** The wound healing assay was employed to determine the migration of HCCLM3^TMEM100+^, HCCLM3^NC^, MHCC97-L^TMEM100+^ and MHCC97-L^NC^ cells. The results showed that closure of HCCLM3^TMEM100+^ and MHCC97-L^TMEM100+^ cells were significantly slower than that of HCCLM3^NC^ and MHCC97-L^NC^ cells (28% versus 89%, 12% versus 80%, respectively, *P* < 0.05). **B.** For the transwell assay, HCCLM3^TMEM100+^, HCCLM3^NC^, MHCC97-L^TMEM100+^ and MHCC97-L^NC^ cells were seeded into the upper chamber of the transwell, respectively. The cells that invaded through the pores to the lower surface of the filter were counted. Our data showed that the numbers of HCCLM3^TMEM100+^ and MHCC97-L^TMEM100+^ cells that passed through the matrigel was significantly less than that of HCCLM3^NC^ cells and MHCC97-L^NC^ cells (*P* < 0.05). **C.** An MTT assay was performed to investigate proliferation of HCCLM3^TMEM100+^, HCCLM3^NC^, MHCC97-L^TMEM100+^ and MHCC97-L^NC^ cells. Every 24 hours the absorbencies of the test well were read and semilogarithm curves were drawn. Only a very subtle increase of proliferation was observed in HCCLM3^TMEM100+^ and MHCC97-L^TMEM100+^ cells as compared with HCCLM3^NC^ and MHCC97-L^NC^ cells (*P* < 0.05). **D.** The cell cycle distribution of HCCLM3 and MHCC97-L cells infected with TMEM100 lentivirus or control vector were analyzed. Our data showed that HCCLM3^TMEM100+^ and MHCC97-L^TMEM100+^cells was arrested at G1 phase as compared with HCCLM3^NC^ and MHCC97-L^NC^ cells (*P* < 0.05). **E.** Colony formation assay was performed to compare the proliferative capacity of HCCLM3^TMEM100+^, HCCLM3^NC^, MHCC97-L^TMEM100+^ and MHCC97-L^NC^ cells. Our data showed that fewer colony formation was observed in HCCLM3^TMEM100+^ and MHCC97-L^TMEM100+^cells as compared with HCCLM3^NC^ and MHCC97-L^NC^ cells (*p* < 0.05). **F.** TMEM100 inhibits HCC cell growth *in vivo*. The model in nude mice was constructed by using HCCLM3 cells infected with control vector or TMEM100 lentivirus. The size of subcutaneous tumors in these two groups were calculated and compared. The size of tumors in overexpressed TMEM100 group was significantly smaller than that of control group (*p* < 0.05). The wound healing assay, transwell assay, MTT assay, cell cycle analysis and colony formation assay were all performed in triplicate.

To determine the biological significance of TMEM100 in HCC metastasis, we first constructed cell lines by using HCCLM3 and MHCC97-L cells infected with control vector or TMEM100 lentivirus. Our data showed that the expression of TMEM100 was significantly overexpressed in HCCLM3 ^TMEM100+^ and MHCC97-L ^TMEM100+^ cells compared to HCCLM3 ^NC^ and MHCC97-L ^NC^ cells (Figure S1, *p* < 0.05). Then, we performed a wound-healing assay and transwell assay using HCCLM3 and MHCC97-L cells. It was noted that ectopic TMEM100 expression significantly suppressed wound healing in the studies with HCCLM3 and MHCC97-L cells (*p* < 0.05, Figure 6A). Transwell assays with matrigel further demonstrated that TMEM100 markedly inhibited the invasive capacity of HCCLM3 and MHCC97-L cells as compared with that of vector-transduced control cells (*p* < 0.05, Figure 6B). To demonstrate the effect of TMEM100 on HCC growth, we performed an HCC cell proliferation assay. As shown in Figure 6C, lentiviral-induced ectopic TMEM100 resulted in a significant decrease in cell proliferation in HCCLM3 and MHCC97-L cells (*p* < 0.05, Figure 6C). We then performed cell cycle analysis and revealed that TMEM100 arrested the cells at G1 phase. Ectopic TMEM100 expression increased the percentage of HCCLM3 cells in G1 phase from 57.0% to 86.6% (*P* < 0.001), but decreased the percentage of cells in S phase and G2/M phase from 11.7% to 19.6% (*P* < 0.05) and 1.7% to 23.4% (*P* < 0.001), respectively (Figure 6D). The same results were obtained in MHCC97-L cells (Figure 6D). Then, colony formation assay was performed to compare the proliferative capacity of HCCLM3^TMEM100+^, HCCLM3^NC^, MHCC97-L^TMEM100+^ and MHCC97-L^NC^ cells. Our data showed that fewer colony formation was observed in HCCLM3^TMEM100+^ and MHCC97-L^TMEM100+^cells as compared with HCCLM3^NC^ and MHCC97-L^NC^ cells (Figure 6E, *p* < 0.05). Furthermore, the effects of TMEM100 expression on tumor cells *in vivo* were also examined. Our data showed that the size of tumors in overexpressed TMEM100 group was significantly smaller than that of control group (Figure 6F, *p* < 0.05). Together, these data support that TMEM100 play an important role in inhibition of HCC metastasis and proliferation.

## DISCUSSION

TMEM100 is a novel gene firstly identified as a transcript from the mouse genome in 2001. TMEM100 transcripts associated with transcripts for activin receptor-like kinase I (ALK1), the gene mutated in hereditary hemorrhagic telangiectasia [15]. The current study demonstrated that the TMEM100 involved in apoptosis [20], angiogenesis [17] and body height [21]. Eisenman ST et al. demonstrated that TMEM100 maybe the enteric nerves novel marker [22].

Recent studies have demonstrated that greater levels of TMEM100 are detected in the transition zone of the prostate when compared to the peripheral zone where the majority of aggressive tumors are detected and it is a possible candidate as a potent tumor suppressor [23]. However, the role of TMEM100 in human HCC still remains unknown and the correlation between its expression and prognosis of patients has not been documented. Thus, we firstly measured TMEM100 mRNA expression in 30 paired HCC and ANLT specimens by RT-PCR analysis. TMEM100 was frequently downregulated in HCC tumor tissues compared to ANLTs. To confirm the expression patterns of TMEM100 mRNA, we conducted semi-quantitative RT-PCR analyses in the same specimens and the consistent results were obtained. Furthermore, we also showed that TMEM100 protein level was decreased in four representative pairs of HCC tissues compared with ANLTs. These results were in line with Frullanti E's research. They found TMEM100 was downregulated in lung cancer and inhibited the proliferation of lung cancer cells [18]. Thus, we speculated that TMEM100 might play a similar role in HCC.

Therefore, we detected the expression of TMEM100 in HCC cell lines with different proliferation potential. We set TMEM100 expression level in the normal liver cell LO2 as a reference. We found TMEM100 expression was downregulated in all HCC cell lines compared with normal liver cell line (L02). Furthermore, we also found that TMEM100 mRNA expression in the HCC cell line HepG2 and SMMC-7721, which with high metastatic and proliferative potential, was very low (0.001 and 0.002), while in HCCLM3 and MHCC97L, which with lower metastatic and proliferative potential, TMEM100 mRNA expression was relatively high (0.40 and 0.17). Similarly, semi-quantitative RT-PCR and Western-blot results are consistent with the results of qRT-PCR. Therefore, we speculate that TMEM100 might inhibit the metastasis and proliferation of HCC cells and act as a tumor suppressor in HCC. Then, to further verify this hypothesis, we analyzed the expression of TMEM100 in ANLTs, well differentiated hepatocellular carcinoma and poorly differentiated hepatocellular carcinoma by immunohistochemistry. Our data showed that reduced expression of TMEM100 was in agreement with the metastatic and proliferative potential of these HCC tissues. Together, these data suggests a potential role for TMEM100 in HCC metastasis and proliferation.

Since TMEM100 maybe a noval tumor suppressor gene in HCC, we further explore the the correlation between TMEM100 expression and the prognosis of patients with HCC. According to the data of immunohistochemistry, we divide all the patients into two groups: the high TMEM100 expression group and the low TMEM100 expression group. Our results demonstrated that HCC patients in the high TMEM100 expression group in general had a better prognosis than those in the low TMEM100 expression group.

Final, we also determined the biological significance of TMEM100 in HCC metastasis and proliferation. We found that TMEM100 significantly inhibited the metastasis and proliferation of HCC cell *in vitro and in vitro*.

In conclusion, TMEM100 is down-regulated in HCC. TMEM100 possesses the potency to suppress HCC growth and metastasis. Therefore, TMEM100 could function as a tumor suppressor in HCC. These data would provide us a wider perspective on HCC intervention/prevention and treatment.

## MATERIALS AND METHODS

### Patients and tissue specimens

A total of 60 HCC specimens were obtained from patients who underwent hepatectomy at the Department of Surgery, Xiangya Hospital of Central South University, Hunan, China, from January 2010 to December 2012. None of the patients in our study received neoadjuvant chemotherapy. These patients included 49 males and 11 females with medianage of 45 years (range, 19-68). Among these patients, 30 matched fresh HCC specimens and adjacent nontumorous liver tissues (ANLTs) were selectively employed for real-time quantitative reverse-transcription polymerase chain reaction (qRT-PCR) and western blot analysis. The diagnosis for each patient was confirmed by histopathology. Prior informed consent was obtained, and the study protocol was approved by the Ethics Committee of Xiangya Hospital.

### Reverse transcription and polymerase chain reaction (RT-PCR)

The expected size of TMEM100 is a 183 bp fragment. Primers synthesized by Invitrogen Company (Shanghai, China) were as follows: forward: 5′-TGCTGTGGTTGTCTTCATCG-3′; and reverse: 5′-CTCTCCCGTCTCTTGGCTTTC-3′. GAPDH gene expression was used as a loading control. The level of TMEM100 mRNA expression was expressed as the relative intensity of the PCR product bands from target sequences compared with that from the GAPDH gene. The PCR experiments were done in triplicate.

### Quantitative real-time reverse transcription polymerase chain reaction (qRT-PCR)

TMEM100 and GAPDH were used as the target gene and internal loading control, respectively. Primers synthesized by Invitrogen Company (Shanghai, China) were as follows: TMEM100-forward: 5′-TGCTGTGGTTGTCTTCATCG-3′; TMEM100- reverse: 5′-CTCTCCCGTCTCTTGGCTTTC-3′; GAPDH-forward: 5-AGAAGGCT-GGGGCTCATTTG-3′; and GAPDH-reverse: 5′-AGGGGCCATCCACAGTCTTC-3′. All amplification reactions were performed in triplicate. The PCR product quality was monitored using post-PCR melt curve analysis. Reactions were carried out in a 96-well plate using the ABI Prism 7300 Real-Time PCR System (Applied Biosystems, Foster City, CA, USA)

### Western blot

Total protein (100 ug) was separated using SDS-PAGE and then transferred onto nitrocellulose membrane(Thermo, USA). The blotted membranes were incubated with goat anti-human TMEM100 polyclonal antibody (Santa Cruz, USA) diluted at 1:200. After washing, the membranes were incubated with a 1:12000 dilution of horseradish peroxidase-linked donkey anti-goat antibody (Santa Cruz, USA). The blots were developed using enhanced SuperSignal West Pico chemiluminescence (Pierce, Rockford, IL, USA). Beta-actin protein was also determined by using the specific antibody (Santa Cruz, USA) as a loading control. All experiments were carried out in triplicate.

### Immunohistochemistry

Formalin-fixed paraffin sections were stained for TMEM100 and Ki-67 using the streptavidin-peroxidase system (Zhong-shan Goldenbridge Biotechnology, Beijing, China). Negative control slides were probed with goat serum followed by the secondary antibody under the same conditions. The expression levels of TMEM100 were scored using the Shimizu criteria according to the percentage of positive hepatocytes: 0, ≤10% positive; 1+, 11% to 25% positive; 2+, 26% to 50% positive; 3+, 51% positive [24]. The protein expression of TMEM100 was thus considered negative if scored 0, and 1+, 2+, and 3+ were considered positive. TMEM100 expression in HCC specimens was also divided into a low-expression group (0 or 1+) and a high-expression group (2+ or 3+).

### Follow-up and prognostic study

All patients were regularly followed up by the same surgical team in our hospital, with surveillance for the recurrence and metastasis by clinical examination, alphafetoprotein levels, and ultrasonography or computed tomography scan every 3 months. The follow-up period was defined as the interval between the date of operation and the date of patient death or last follow-up. Deaths from other causes were treated as censored cases. The disease-free survival was defined as the length of time after hepatic resection for HCC during which a patient survives with no evidence of HCC. Thirteen conventional variables together with TMEM100 expression were tested in all 90 patients: gender, age, HBsAg, AFP, cirrhosis, Child-Pugh Score, tumor size, capsular formation, tumor nodule number, TNM stage, BCLC stage, Edmondson-Steiner stage and venous invasion.

### Cell lines and cell culture

HepG2 cell lines were purchased from the ATCC (Manassas, Va). LO2, SMMC-7721, MHCC97-L, MHCC97-H, and HCCLM3 cell lines were purchased from the Liver Cancer Institute of Fudan University. All cell lines were routinely cultured in Dulbecco's modified Eagle medium supplemented with 10% fetal bovine serum and antibiotics at 37 C with 5% CO2.

### Wound healing and transwell assay

For the wound healing assay, cells were seeded into 35-mm dishes precoated with fibronectin. When cells reached 100% confluence, a scratch was made with a pipette tip. The cells were then cultured for another 48 hours and a micrograph was taken every 24 hours. For the Transwell assay, approximately 1×10^5^ cells were placed into the upper chamber of the insert with Matrigel (BD Biosciences, Woburn, Mass). The cells were cultured in serum-free medium in 5% CO2 for 48 hours, then cells in the upper chamber were removed with cotton swabs and stained with a solution containing 0.1% crystal violet and 20% methanol. The number of cells that adhered to the lower membrane of the inserts was counted. For each experimental group, the invasion assay was performed in triplicate, and 3 random fields were chosen for cell number quantification.

### Proliferation assays

The proliferation of HCCLM3 was assessed using the MTT method. Briefly, HCCLM3 cells were plated in 48-well plates at 2×10^4^ per well. After incubation for 24 h, 20μL MTT with 5 mg/mL concentration was added to the medium and cultured for another 4 h. The medium was then abandoned and 150μL of DMSO was added into each well, rocked for 10 min and the absorbencies of each well were read using a microplate reader at a wavelength of 490 nm. Semi-logarithmic curves were drawn with cell viability using Microsoft Excel 2003 software (Microsoft Corporation, Redmond, WA, USA).

### Mouse model

The model in nude mice was constructed as described before [25]. Briefly, 5 × 10^6^ HCCLM3 cells were injected subcutaneously into the left upper flank regions of nude mouse (3-4 weeks of age, male, BALB/c). The subcutaneous tumor tissues were removed one month later. The size for tumors was calculated as follows: tumor volume (mm3) = (L × W^2^)/2, where L = long axis and W = short axis. [26] All animal studies were conducted in the Animal Institute of CSU according to the protocols approved by the Medical Experimental Animal Care Commission of CSU.

### Statistical analysis

Statistical analysis was performed using the SPSS (version 17.0, Chicago, IL). Data for TMEM100 expression in fresh specimens were analyzed using the Mann–Whitney U-test. Fisher's exact test was used for statistical analysis of categorical data, whereas independent t tests were used for continuous data. Spearman correlation test was used for analyzing the correlations between TMEM100 expression level and the clinical and pathological variables. Survival curves were constructed using the Kaplan-Meier method and evaluated using the log-rank test. The cox proportional hazard regression model was established to identify factors that were independently associated with overall survival. In any case, *P* < 0.05 was considered with statistical significance.

## SUPPLEMENTARY MATERIAL FIGURE



## Abbreviations

HCC: hepatocellular carcinoma
SLHCC: solitary large hepatocellular carcinoma
NHCC: nodular hepatocellular carcinoma
SHCC: small hepatocellular carcinoma
qRT-PCR: Real-time quantitative PCR
