# Supplementary material for: Novel roles of TMEM100: inhibition metastasis and proliferation of hepatocellular carcinoma
Source: Oncotarget. 2015 Apr 29;6(19):17379–90. doi: 10.18632/oncotarget.3954 (PMC4627315; doi:10.18632/oncotarget.3954)
Supplement: Supplementary file 1 [file oncotarget-06-17379-s001.pdf]

Novel roles of TMEM100: inhibition metastasis and proliferation of hepatocellular carcinoma

Supplementary Material

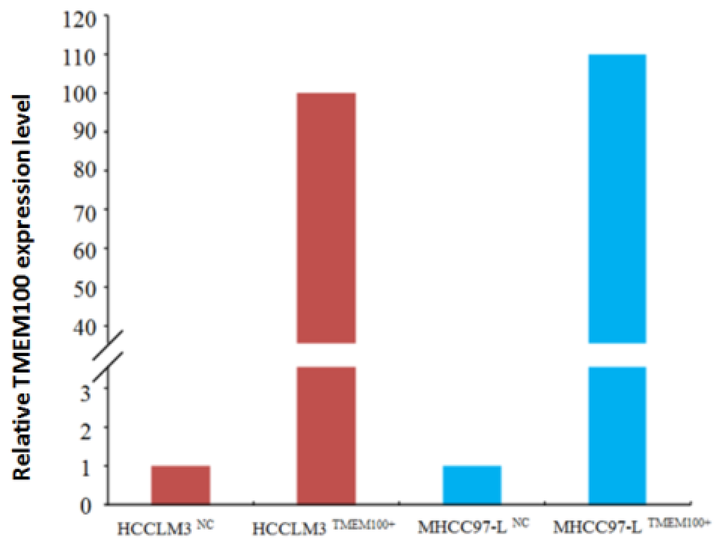

Supplementary Figure 1: The relative expression level of TMEM100 in HCCLM3 ^{NC}, HCCLM3 $^{TMEM100+}$, MHCC97-L ^{NC} and MHCC97-L $^{TMEM100+}$ cells. The expression of TMEM100 was significantly overexpressed in HCCLM3 $^{TMEM100+}$ and MHCC97-L $^{TMEM100+}$ cells compared to HCCLM3 ^{NC} and MHCC97-L ^{NC} cells (p<0.05).
